# An Automated Imaging-Based Screen for Genetic Modulators of ER Organisation in Cultured Human Cells

**DOI:** 10.3390/cells13070577

**Published:** 2024-03-26

**Authors:** M. Elena Garcia-Pardo, Jeremy C. Simpson, Niamh C. O’Sullivan

**Affiliations:** 1 UCD School of Biomolecular and Biomedical Science, UCD Conway Institute, University College Dublin, 4 Dublin, Ireland; 2 Cell Screening Laboratory, UCD School of Biology and Environmental Science, University College Dublin, 4 Dublin, Ireland

**Keywords:** endoplasmic reticulum, high-content imaging, automated image analysis, siRNA screen, human cells

## Abstract

Hereditary spastic paraplegias (HSPs) are a heterogeneous group of mono-genetic inherited neurological disorders, whose primary manifestation is the disruption of the pyramidal system, observed as a progressive impaired gait and leg spasticity in patients. Despite the large list of genes linked to this group, which exceeds 80 loci, the number of cellular functions which the gene products engage is relatively limited, among which endoplasmic reticulum (ER) morphogenesis appears central. Mutations in genes encoding ER-shaping proteins are the most common cause of HSP, highlighting the importance of correct ER organisation for long motor neuron survival. However, a major bottleneck in the study of ER morphology is the current lack of quantitative methods, with most studies to date reporting, instead, on qualitative changes. Here, we describe and apply a quantitative image-based screen to identify genetic modifiers of ER organisation using a mammalian cell culture system. An analysis reveals significant quantitative changes in tubular ER and dense sheet ER organisation caused by the siRNA-mediated knockdown of HSP-causing genes *ATL1* and *RTN2*. This screen constitutes the first attempt to examine ER distribution in cells in an automated and high-content manner and to detect genes which impact ER organisation.

## 1. Introduction

Hereditary spastic paraplegias (HSPs) comprise a group of inherited neurological disorders characterised by the progressive degeneration of the longest motor neurons (MNs) in the corticospinal tract [1]. HSPs are single-gene inherited disorders which present a vastly heterogenous genetic landscape, encompassing over 80 loci, reviewed in [2]. Despite the increasing number of genes linked to HSP, the functional pathways in which these proteins operate are relatively few, pointing to key molecular mechanisms underpinning neurodegeneration in these disorders [1]. The most frequent cause of HSP are mutations in genes that encode proteins which shape and localise to the endoplasmic reticulum (ER). These include the following: the atlastin (ATL) family of GTPases that drive the homotypic fusion of ER tubules necessary for the formation of the tubular ER network [3]; the reticulons (RTNs) and receptor expression-enhancing proteins (REEP)/DP1/Yop1p families that generate and maintain the high membrane curvature found in ER tubules and sheet edges [4,5,6]; and the microtubule-severing ATPase Spastin (*SPG4*) which regulates ER distribution and calcium handling [7,8]. Taken together, the disruption in ER organisation is thought to be a central pathogenic mechanism underpinning HSPs.

The disturbance to several other cellular events are suggested to contribute to pathogenesis in HSPs, particularly lipid homeostasis, mitochondrial organization, and intracellular trafficking. The disruption in lipid biogenesis and storage in lipid droplets (LDs) are a common feature of HSPs. Numerous subtypes of HSP are caused by mutations encoding ER-resident enzymes involved in lipid synthesis, e.g., the phospholipase PNPLA6 (*SPG39*) [9,10], LD production, e.g., Seipin [11,12], and LD turnover, e.g., Spartin (*SPG20*) [13,14]. LD defects have also been reported in cell and animal models of HSP with mutations in ER-shaping proteins [15,16]. Another common feature of HSP-causing mutations and model systems is the regulation of mitochondrial function and organisation. A number of HSP gene products are directly involved in ATP synthesis and mitochondrial network maintenance (reviewed in [17]). Additionally, both patient-derived samples and model systems of HSP frequently display a disrupted mitochondrial morphology consistent with altered mitochondrial fission/fusion dynamics [18,19,20,21]. Lastly, intracellular trafficking processes, including the endolysosomal and autophagic pathways, are strongly associated with HSP (reviewed in [22]). The HSP-causing genes *SPG11* and *SPG15* encode proteins, spatacsin, and spastizin, respectively, that mediate autophagic lysosomal reformation [23], and cell and animal models of *SPG11*/*SPG15* display endolysosomal abnormalities [24,25]. Furthermore, several cellular models of HSP associated with *SPG4*, REEP1 (*SPG31*), and Strumpellin (*SPG8*) show defects in mannose 6-phosphate receptor (*M6PR*) sorting and an abnormal lysosomal morphology [26]. While there are well-established physical links between the ER and LDs, the mitochondria, and the endolysosmal system [27], it is not clear whether the loss of HSP-causing genes involved in LDs, the mitochondria, or the endolysosomal system directly disrupt ER organisation.

The ER is a highly complex organelle reaching across the cell from the nuclear envelope to the plasma membrane. Its complex architecture defines functionally and structurally distinct domains, which enable the ER to conduct a plethora of very distinct functions [28]. The main structural domains that make up the ER are the ribosome-studded sheets of “rough” ER (RER), which are stacked perinuclearly, and a network of tubules spreading throughout the cell’s cytoplasm to the plasma membrane [29,30]. The expansive and complex structure of the ER has meant that developing methods to quantitatively compare changes in its organisation across cell populations has proven challenging. Until now, the majority of studies reporting on ER morphology have been qualitative, i.e., describing the organelle as ‘normal’ or ‘abnormal’. Qualitative studies are limited to the visual detection of gross changes, such as may occur under high levels of stress or cell toxicity, but often fail to detect more subtle, yet physiologically relevant, changes, like those observed in HSP. Recently, we have developed a robust automated pipeline to quantitatively assess ER structures [31]. We have now applied this approach to evaluate how neurodegeneration-associated genes impact ER organisation in vitro using gene-silencing technology. This study constitutes the first attempt to examine the ER morphology in cells to screen for genes which disrupt key features of ER organisation.

## 2. Materials and Methods

### 2.1. Cell Culture

Human osteosarcoma epithelial cells (U-2 OS), which were genetically modified to express Sec61β-mEmerald [32] and kindly provided by Craig Blackstone from Massachusetts General Research Institute, were cultured in Dulbecco’s modified Eagle’s medium (DMEM) supplemented with 1 g/L glucose (Lonza, Basel, Switzerland, LZBE12-707F), 10% heat-inactivated fetal calf serum (Sigma-Aldrich, MO, USA F9665), 0.8% L-glutamine (Thermo Fisher Scientific, Waltham, MA, USA, 25030024), and 10 μg/mL (or 0.1%) G-418 (Thermo Fisher Scientific, 10131035). The cells were maintained in a humidified incubator at 37 °C with 5% CO_2_.

### 2.2. Multiplex siRNA Screen

#### 2.2.1. Preparation of Solid-Phase Transfection siRNA Assay Plates

Plates for siRNA multiplex assays were prepared following the protocol for solid-phase transfection adapted from Galea and Simpson [33] to 96-well format. All the steps of the protocol were carried out in a sterile laminar flow cabinet. Briefly, deep-well 96-well v-shaped mixing plates were coated with a 13.7% sucrose (Sigma-Aldrich, S0389) OptiMEM Reduced-Serum Medium (Thermo Fisher Scientific, 31985062) solution combined with Lipofectamine 2000 transfection reagent (Thermo Fisher Scientific, 11668-019) at a ratio of 1:1.125 Opti-MEM/sucrose:Lipofectamine. siRNA (6 μM) each from a siRNA custom library (Supplementary Table S1; Thermo Fisher Scientific) were added to each well of the mixing plate, sealed, centrifuged at 2000 rpm for 3 min, and incubated at room temperature for 20 min to allow for transfection complex formation. Finally, a solution of 0.1% human plasma fibronectin (Sigma-Aldrich, F0895) and 0.08% bovine skin gelatin (Sigma-Aldrich, G9391) in RNase-free water (Thermo Fisher Scientific, 7732-18-5) was added to the transfection mixture in the mixing plate. Using a multichannel pipette, 50 μL of this mix was transferred to assay plates (CellCarrier Ultra 96-well optically clear plates (PerkinElmer, Shelton, CT, USA, 6055300)) and vacuum-dried for 6 h at 37 °C using a centrifugal vacuum concentrator miVac Quattro (Genevac, Ipswich, UK). Plates were stored in a sealed container with desiccating agent for up to one year.

#### 2.2.2. siRNA Screen

2200 cells were seeded per well on plates with desiccated transfection mixture allowing for growth and siRNA transfection for 72 h. After concluding the experiments, cells were stained by incubation at 37 °C for 10 min with a pre-warmed solution consisting of Hoechst 33342 (10 μM; Thermo Fisher Scientific, 62249) and CellMask™ Deep Red PM stain (1:10,000 dilution; Thermo Fisher Scientific, C10046) in DMEM medium. This staining served to label the nuclei and plasma membrane (PM), respectively. Subsequently, the cells were washed with PBS buffer at pH 7.4, fixed with 4% (*w/v*) paraformaldehyde (PFA) for 15 min at room temperature, subjected to three additional washes with PBS buffer, and, finally, imaged.

#### 2.2.3. Automated Confocal Imaging and ER Analysis

Confocal imaging was performed using the Opera Phenix High-Content Screening System (PerkinElmer), capturing 5 optical slices spaced 0.5 μm apart across 7 × 7 fields of view, with a 10% overlay. A 63×/1.15 NA water-immersion objective was utilised, providing an effective xy resolution of 0.28 μm. The acquired images were analysed using an automated pipeline developed with Harmony^®^ v 4.8 high-content analysis software (PerkinElmer), running on a computer equipped with an Intel Xeon CPU operating at 3.60 GHz, 80 GB RAM, and a 64-bit Windows 10 Pro operating system, as previously described in [31]. In brief, the Harmony^®^ image analysis pipeline quantifies polygonal region areas, which represent the spaces enclosed by ER tubules within the peripheral tubular ER network. Additionally, the pipeline computes the percentage of the cell occupied by higher-intensity pixels corresponding to the dense ER in the perinuclear cell area.

#### 2.2.4. Liquid-Phase siRNA Transfection

Validation of screen results was performed using liquid-phase siRNA transfection. Cells were plated into standard 24-well plates or 96-well imaging plates (PerkinElmer, 6055300). After 24 h, cells were transfected with siRNAs (Supplementary Table S2; Thermo Fisher Scientific) mixed in the ratio of 1 pM siRNA: 0.1 μL RNAiMAX in OptiMEM and incubated for 72 h. Cells in 24-well plates were used for RNA extraction. Cells in 96-well imaging plates were stained, fixed, imaged, and analysed as described above.

### 2.3. Quantitative PCR

Total RNA was purified using 250 μL TRIzol^TM^ reagent (Thermo Fisher Scientific, 15596026) on ice according to the manufacturer’s protocol. cDNA synthesis was carried out utilising the SuperScript III First Strand Kit (Thermo Fisher Scientific, 18080044) with DNase-treated RNA. Real-time quantitative PCR (qPCR) analysis was performed using Fast SYBR^®^ Green Real-Time PCR Master Mix (Applied Biosystems, Waltham, MA, USA, 4385612) and conducted on a 7500 FAST real-time PCR system (Thermo Fisher Scientific). The expression of the GAPDH gene served as the reference for each sample. Primer pairs for each target can be found in Supplementary Table S3. Three biological replicates were performed, and the relative gene expression was determined using the −ΔCt method, with mRNA levels from siRNA-treated cells normalised to those treated with non-targeting scrambled siRNA control (NTC) reagents.

### 2.4. Statistical Analysis

All data were statistically analysed using Prism 8 (GraphPad Software, Inc., La Jolla, CA, USA). For analysis of ER morphological readouts for the screen, 70 cells per well were randomly selected with 3 independent wells per target siRNA and 9 independent wells of non-targeting scrambled siRNA control (NTC). Statistical analysis was conducted using one-way ANOVA and Dunnett’s post-tests. Unless otherwise specified, statistically significant differences from control are indicated throughout all graphs as * *p*  <  0.05, ** *p*  <  0.01, *** *p*  <  0.001, and **** *p*  <  0.0001. *p*  >  0.05 is indicated as ns (not significant).

## 3. Results and Discussion

**Figure 1:**
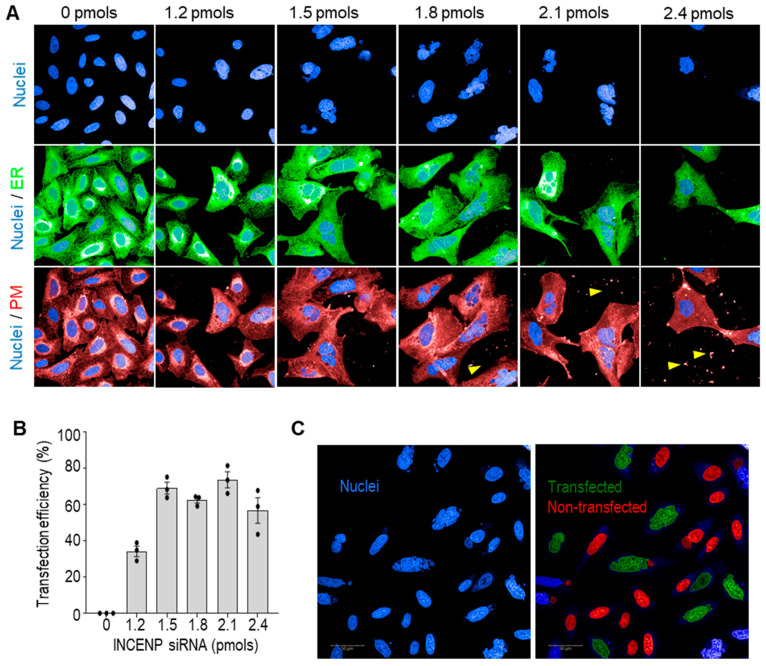
Optimisation of siRNA solid-phase transfection efficiency using the visible phenotypic siRNA *INCENP*. (**A**) Representative images of Sec61β-mEmerald (ER) expressing U-2 OS treated for 72 h with different amounts of *INCENP* siRNA via solid transfection. Cells treated with 1.8, 2.1, and 2.4 pmols exhibited release of materials to the media stained with CellMask plasma membrane (PM) stain (yellow arrowheads) (**B**) Quantification of transfected cells showing *INCENP* phenotype. Data are expressed as mean ± SEM (0–2.1 pmol treatments *n* ≥ 75 cells from 3 independent experiments; 2.4 pmol treatment reduced cell proliferation *n* ≥ 17 cells from 3 independent experiments). (**C**) Example of algorithm detection of *INCENP*-depleted cells (green) used to assess transfection efficiency. Objects on image borders are excluded from the analysis.

We set out to use our automated imaging-based pipeline to screen for genetic modifiers of ER organisation in human cells. To ensure the accuracy and reproducibility of the results, we used a solid-phase transfection method [33,34]. This approach offers advantages to liquid-based forward transfection in large-scale screening, as it allows the uniform preparation of all plates beforehand, in order to minimise the variation of treatments across replicates. We optimised the protocol for the 96-well format, using siRNA targeting the inner centromere protein (*INCENP*) to monitor the transfection efficiency vs toxicity: 1.5 pmols of siRNA per well resulted in the highest transfection efficiency without evident signs of cell toxicity (Figure 1). Cells in wells treated with >1.5 pmols siRNA showed signs of toxicity and reduced proliferation. We, therefore, treated cells in the screen with 1.5 pmols siRNA per well for the screen. *INCENP* depletion inhibits complete nuclear fission during cell division, resulting in a visible phenotype observed as ‘multilobed’ nuclei of the enlarged nuclear area and irregular circularity [35]. We designed a short analysis pipeline that quantifies the proportion of cells presenting this phenotype (Figure 1C). This was used to verify consistent siRNA transfection efficiency on all plates used in the screen.

**Figure 2:**
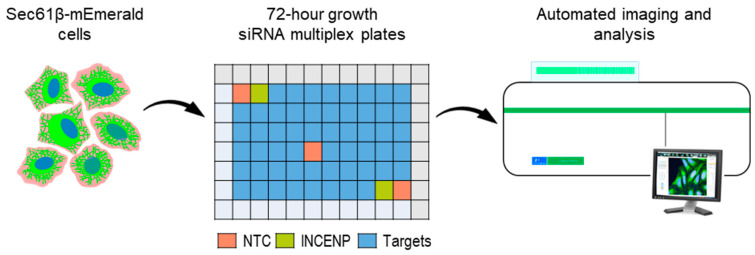
Sec61β-mEmerald expressing U-2 OS cells were seeded in plates containing desiccated siRNA transfection mixture, grown for 72 h, and imaged using the Opera Phenix automated microscope.

The screen was designed in a 96-well format, based on a custom library of siRNAs targeting 55 candidate genes. All outer wells contained medium-only ‘untreated’ cells to prevent ‘edge effect’ artefacts [36] (Figure 2). The 55 candidate genes were selected to include genes encoding proteins proposed to directly function in ER organisation (e.g., ER-shaping proteins: Atlastin 1 (*ATL1*) and Reticulons 2 and 3 (*RTN2* and *RTN3*)) or function (e.g., ER stress response proteins *TMEM33* and *PERK*/*EIF2AK3*) (Supplementary Table S1). We also examined genes encoding proteins with roles in the functions related to HSP including the regulation of mitochondrial organisation (e.g., mitochondrial fusion proteins *MFN1* and *MFN2*), lipid regulation (e.g., lipid droplet regulating proteins *Seipin* and *ORP8*), and cell trafficking (e.g., vesicle transport proteins *RAB1A*, *RAB2A,* and *RAB7A*). Representative genes from these pathways were selected as they functionally overlap with the ER via ER contacts [27]; however, as yet, it is unclear whether the loss of these genes might directly alter ER organisation in human cells. Each plate also included three non-targeting scrambled siRNA control (NTC) wells in different positions, and two wells of siRNA targeting *INCENP*, to verify that the siRNA transfection efficiency of all plates in the screen was at least 65%. U-2 OS cells stably expressing Sec61β-mEmerald were plated on the desiccated transfection mixture for 72 h before being fixed and imaged. To ensure that treatment with oligonucleotides generally did not significantly impact the ER morphology, we analysed and compared the polygon area and % dense ER in NTC and untreated cells and found no significant changes in either metric of ER organisation.

**Figure 3:**
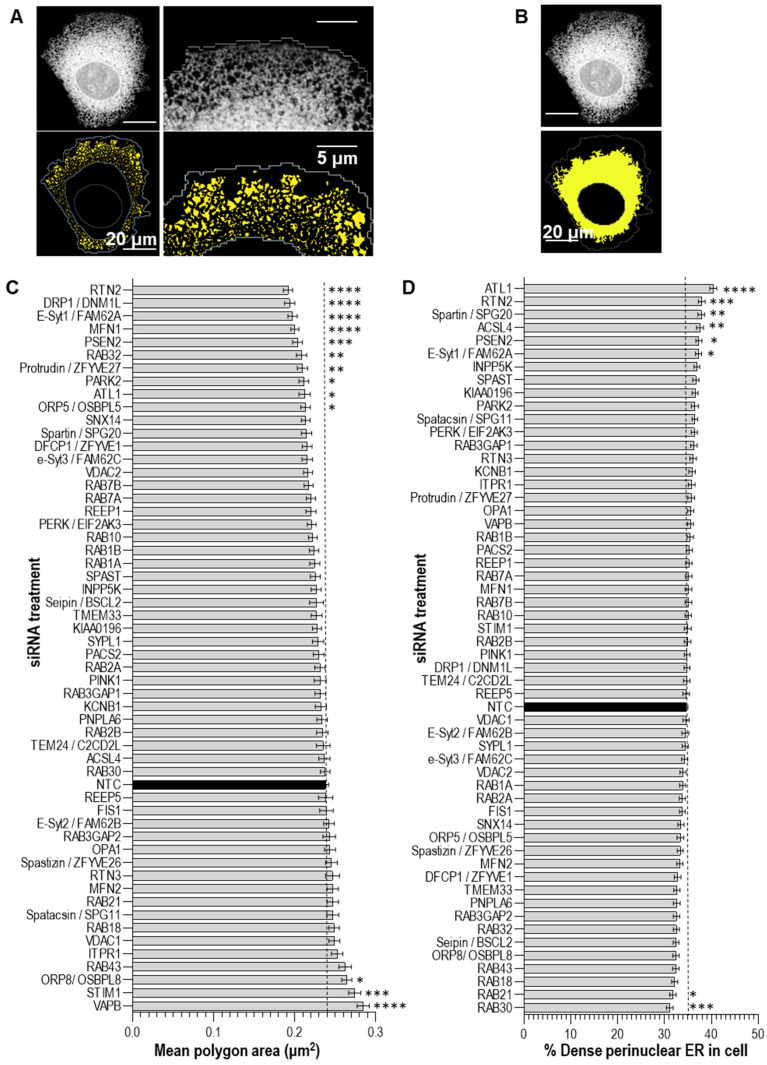
Altered ER organisation assay in Sec61β-mEmerald expressing U-2 OS cells. Cells were treated with siRNAs for 72 h, imaged by automated confocal microscopy, and analysed using an automated image analysis pipeline. Shown are representative confocal images of a Sec61β-mEmerald expressing cell (top panels) and illustrating the polygons (**A**) and perinuclear ER (**B**) as identified by the pipeline (yellow; lower panels). Graphs represent mean ± SEM tubular ER polygon area (**C**) and % dense perinuclear ER (**D**) within each cell. *n* = minimum of 210 cells from 3 independent experiments. Values for control (NTC) siRNA treated cells shown in black. Statistical significance compared to values in NTC control were assessed by one-way ANOVA with Dunnett’s post-hoc tests. * *p*  <  0.05, ** *p*  <  0.01, *** *p*  <  0.001, and **** *p*  <  0.0001.

**Figure 4:**
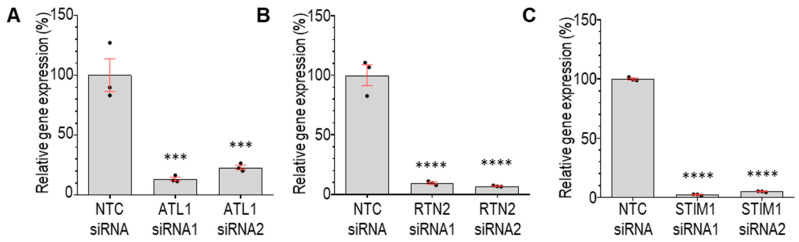
Knockdown efficiency of siRNAs. Graphs represent mean ± SEM real-time PCR analysis of cells treated with non-targeting control (NTC) siRNA or siRNA sequences targeting *ATL1* (**A**), *RTN2* (**B**), or *STIM1* (**C**) for 72 h. Statistical analysis consists of one-way ANOVA and Tukey’s multiple comparisons tests. *n* = 3 biological replicates. *** *p*  <  0.001, and **** *p*  <  0.0001.

**Figure 5:**
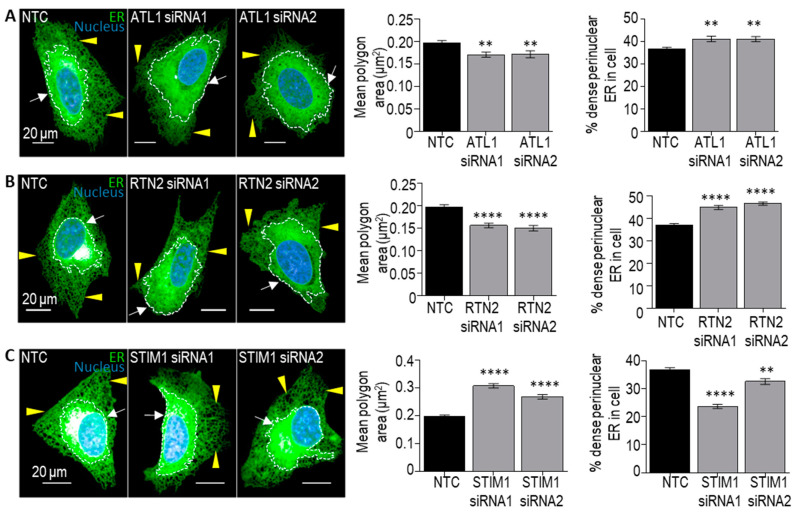
Validation of genetic modifiers of ER network organisation. Representative images of ER (green) distribution changes occurred in U-2 OS cells expressing Sec61β-mEmerald upon 72 h incubation with siRNAs targeting *ATL1* (**A**), *RTN2* (**B**), or *STIM1* (**C**). Dense ER (discontinuous white line, white arrow). Tubular ER polygons (yellow arrowheads). Nucleus (blue). Scale bars = 20 μm. Graphs represent quantification of changes in polygon region area and % of dense ER in cell for each treatment. Data are expressed as mean ± SEM of tubular ER polygon area and % dense perinuclear ER within each cell as indicated. *n* = minimum of 210 cells from 3 independent experiments. Statistical significance compared to values in NTC control were assessed by one-way ANOVA with Dunnett’s post-hoc tests. ** *p*  <  0.01 and **** *p*  <  0.0001.

Our screen focused on two readouts of ER organisation: (1) the area of the polygon regions between ER tubules within the periphery of the cell (Figure 3A), and (2) the % of the cytoplasm occupied by dense perinuclear ER (Figure 3B) [31]. An increase in the polygon area from control indicates that the siRNA results in a less dense tubular ER network. An increase in the % dense perinuclear ER in a cell indicates that the siRNA results in an expansion of the ribosome-studded sheet ER, while, vice versa, a decrease in the % dense perinuclear ER in a cell indicates that the siRNA results in a contraction of the sheet ER. We found that, while the majority of siRNAs tested did not significantly alter these readouts of ER organisation, treatment with several siRNAs did significantly alter ER organisation compared to NTC-treated cells (Figure 3C,D). In particular, the knockdown of the genes encoding ER-shaping proteins *ATL1* and *RTN2* were found to result in a significant reduction in the polygon area from control, indicating that the knockdown of these genes results in a more densely packed tubular ER network, and an expansion of the dense sheet ER. Interestingly, the loss of function mutations in both of these genes give rise to the neurodegenerative disorder hereditary spastic paraplegia (HSP) (subtypes SPG3A and SPG12, respectively). We, therefore, used alternative siRNA sequences in independent experiments to determine whether these changes could be validated. Similar to the results from the screen, the efficient knockdown of *ATL1* (SPG3A) with two independent siRNAs (Figure 4A) also resulted in a significant reduction in the tubular ER polygon area and a significant expansion of the dense sheet ER in cells (Figure 5A), compared to NTC-treated controls. Similarly, the efficient depletion of *RTN2* (SPG12) with two independent siRNAs (Figure 4B) significantly reduced the area of tubular ER polygons and expanded the dense sheet ER (Figure 5B) in agreement with the findings from the screen. These changes in ER organisation may reflect a shift from tubular to ribosome-studded sheet ER within cells deficient in *ATL1* or *RTN2*; however, further studies would be required to confirm this. These findings support the model that disrupted ER organisation contributes to the pathogenesis of neurodegeneration in these forms of HSP.

The knockdown of three genes was found to result in a less dense tubular ER network, by significantly increasing the mean polygon area: *VAPB*, *STIM1,* and *ORP8* (Figure 3C). The proteins encoded by these genes all localise to ER-contacts with other organelles, including ER-mitochondrial contacts (*VAPB* and *ORP8*), ER-microtubule contacts (*STIM1*), and ER-plasma membrane contacts (*ORP8*), so we were interested in studying these further. Neither of the siRNAs that we tested induced the efficient knockdown of *VAPB* expression and the knockdown of *ORP8* did not significantly alter either readout of ER organisation. However, the efficient knockdown of *STIM1* (Figure 4C) results in significantly enlarged tubular ER polygons (Figure 5C), confirming the less dense tubular ER network detected in the screen. *STIM1* encodes stromal interaction molecule 1, an ER-resident Ca^2+^ sensor that interacts with growing microtubules (MTs) co-ordinating the extension or retraction of ER tubules along with MT growth or contraction [37,38]. Interestingly, the overexpression of *STIM* has recently been shown to recue axonal defects in patient-derived cells carrying pathogenic variants in the gene encoding the ER-shaping protein Spastin (*SPAST*) mutations which give rise to HSP subtype SPG4 [8]. This further supports the idea that our screen is reliably detecting genetic modulators of ER organisation in human cells.

## 4. Conclusions

Here, we report the first automated image-based screen of genetic modifiers of ER organisation within adherent cells, specifically quantifying tubular ER polygons and dense perinuclear ER. We screened 55 ER, lipid, mitochondrial, and trafficking-associated genes and found that siRNAs targeting HSP-causing ER-shaping proteins (including *ATL1* and *RTN2*) result in the largest alterations in the ER network organisation, consistent with a central role for the disrupted ER organisation in the pathogenesis of HSP.

## Data Availability

Data are contained within the article and Supplementary Materials.

## Supplementary Materials

The following supporting information can be downloaded at: https://www.mdpi.com/article/10.3390/cells13070577/s1, Table S1: siRNA sequence custom library plate used in screening assay. Table S2: siRNA sequences of siRNAs in validation knockdown studies. Table S3: Primer sequences used for quantitative PCR.
