# Supplementary material for: An Automated Imaging-Based Screen for Genetic Modulators of ER Organisation in Cultured Human Cells
Source: Cells. 2024 Mar 26;13(7):577. doi: 10.3390/cells13070577 (PMC11011067; doi:10.3390/cells13070577)
Supplement: Supplementary file 1 [file cells-13-00577-s001.zip › cells-2889456-supplementary.pdf]

	Gene	Well/s	Gene ID	siRNA ID	Antisense siRNA Sequence
**Plate controls**	Non-targeting siRNA	B2; E6; G11	-	s813	-
	INCENP	B3	3619	s7422	*UAGUUAUCUGGGUUGAUCUtg*
	INCENP	G10	3619	s7423	*UCUGAUAUGAGGUACAAGCtg*
**ER organisation and function**	RTN2	B4	6253	s12381	*UAUUGGUCGAUCUGAGCCUgg*
	ATL1	B9	51062	s27328	*UGUAUUGAGCUGAUCAUUGtg*
	Protrudin; ZFYVE27	C5	118813	s42248	*UAUCGAACACCAUCACCUGca*
	PSEN2	C8	5664	s11294	*UAAAAGAUGAGCCCGAACGtg*
	REEP1	B6	65055	s35169	*UUAGUUCAUAAUAGAAUGGaa*
	PERK; EIF2AK3	C7	9451	s18102	*UCUUGUUCCAUUUCGUCACta*
	Stumpellin; KIAA0196	C3	9897	s19173	*UCUCGGGAUAGUUGGAUGGtc*
	PACS2	C4	23241	s23369	*UUGUUGAACAGGUCUCUCCag*
	REEP5	B7	7905	s15454	*UUCACGGUAGCUUUCUUCGct*
	TMEM33	C6	55161	s30316	*UGCUUAACUGGAAGUGUGGta*
	RTN3	B5	10313	s20161	*UUCCGUUAAAAACAGCACCaa*
	Kv2.1; KCNB1	D5	3745	s7700	*UUUGUUAGGAGACAAGUGGtt*
	SPAST	B8	6683	s13348	*UAUAAGGUUAGCAAGGUUGct*
	IP3R; ITPR1	C2	3708	s7631	*UCCAUAGUAACGGUUGACCag*
	STIM1	G9	6786	s13561	*AACGGUUCUGGAUAUAGGCaa*
	VAPB	B10	9217	s17623	*UAAGGUCUUCCGGUUUUGCct*
**Mitochondrial organisation**	MFN1	D10	55669	s31218	*AUAACUGAGAUCAAAUUUCtt*
	DRP1; DNM1L	E2	10059	s19559	*UUAGGAUUACUGAUGAACCga*
	VDAC2	E7	7417	s14771	*UCUUGUAAGAAGACUUGAUtt*
	VDAC1	E5	7416	s14768	*UAGUGUAUUGUCGGUAUUCca*
	FIS1	E3	51024	s27265	*AGACGUAAUCCCGCUGUUCct*
	PARK2	D9	5071	s10043	*UUGCGAUCAGGUGCAAAGCta*
	MFN2	D11	9927	s19260	*AAACUUGUCAAUCCAGCUGtc*
	OPA1	E4	4976	s9850	*UAUCAGAUAUGGAUCGGUCtt*
	PINK1	D8	65018	s35166	*AUAGUUCUUCAUAACGAGGaa*
**Lipid regulation**	Spartin; SPG20	C9	23111	s23055	*UGACGUACAUCCUUAUUGCct*
	ACSL4	B11	2182	s5000	*UCCAUAGCAGGAUUAUUGCag*
	SNX14	D6	57231	s32927	*UCUAGGUUCUGCAAAUUUCtg*
	DFCP1; ZFYVE1	D7	53349	s28712	*UAUUUUGCGAGACCCAUCCag*
	ORP5; OSBPL5	E11	114879	s376	*UCCUUGAUAAACACGUCCCtg*
	E-Syt1; FAM62A	F2	23344	s23605	*AUAGCUGAUGUUCAAGUCCag*
	E-Syt3; FAM62C	F4	83850	s38209	*UAAAGGUAAAGGUCCUCAGgt*
	PNPLA6	C11	10908	s21441	*UUCCGCAUAAUCUUCCGGCca*
	TEM24; C2CD2L	D4	9854	s19071	*AUCAGUUCCUCAAUUGUGGag*
	INPP5K	E9	51763	s28636	*AUAUAUGUCAAGAUUGAGGtt*
	Seipin; BSCL2	C10	26580	s25557	*UAAGGUAACACGAUACGGCtg*
	ORP8; OSBPL8	E8	114882	s41690	*UACCGCUAAGGCAAAAUCCat*
	E-Syt2; FAM62B	F3	57488	s33136	*ACCUUAACACCAUUGAUCCtg*
**Cell trafficking**	RAB10	F6	10890	s21390	*UACAACUCUUUUGUCGUCCat*
	RAB7A	G6	7879	s15442	*UCAAAUACCAGAACGCAGCag*
	RAB32	F5	10981	s21618	*UAGUAUACUCGGGUCAUGUtg*
	SYPL1	E10	6856	s13691	*AAGUGUAACAACAAAGUCUat*
	RAB1B	F10	81876	s117	*UCCGCUUUUUGAUUUCAGCag*
	RAB7B	G7	338382	s50333	*UCUGGUACCUCGACAGAGCcc*
	RAB2A	G4	5862	s11660	*UAAUGUCAAAGACUCCUUCtt*
	RAB3GAP1	G2	22930	s22700	*UUUUCGAACUCUAACAGACca*
	RAB2B	G5	84932	s39689	*UGAUAGAACGGAAGGAUUCtt*
	Spatacsin; SPG11	D2	80208	s37057	*UGAACAUCUGCUUAUGUCCtg*
	RAB1A	F9	5861	s11658	*UCAGAUCACAUUUGUUCCCta*
	RAB3GAP2	G3	25782	s24474	*UUACUUGCAACUGCUAGUGca*
	RAB30	F11	27314	s26136	*UAAUCAUAAAAUCAACUCCaa*
	Spastizin; ZFYVE26	D3	23503	s23943	*AUAGAGUAAAACACCAUUCct*
	RAB21	G8	23011	s22823	*UCUGUAGUAAAUUGGACCCaa*
	RAB43	F8	339122	s50452	*UCAGGCACCAAGUCUGUGGgt*
	RAB18	F7	22931	s22703	*UUUAGCCUUAUUUCCAUCCac*

Supplementary Table 1. siRNA sequence custom library plate used in screening assay.

Gene	Gene ID	siRNA Seq.	siRNA ID	Antisense siRNA Sequence
**RTN2**	6253	'siRNA1'	s12381	*UAUUGGUCGAUCUGAGCCUgg*
**RTN2**	6253	'siRNA2'	s12382	*UGGUUUGACUCUUCUCAUUtt*
**ATL1**	51062	'siRNA1'	s27328	*UGUAUUGAGCUGAUCAUUGtg*
**ATL1**	51062	'siRNA2'	s27328;<br>s27329;<br>s27330 (1:1)	*AUAGAUCUUUAUAUAAGCCtt*<br>*GGAGUUUCCCAUACGAAUUtt*<br>*AAUUCGUAUGGGAAACUCCag*
**STIM1**	6786	'siRNA1'	s13561	*AACGGUUCUGGAUAUAGGCaa*
**STIM1**	6786	'siRNA2'	s13561;<br>s13562;<br>s13563 (1:1)	*UUCGGCAAAACUCUGCUGCag*<br>*GGAUUUGACCCAUUCCGAUtt*<br>*AUCGGAAUGGGUCAAAUCCct*

Supplementary Table 2. siRNA sequences of siRNAs in validation knockdown studies.

RTN2	Forward: 5'- TCGTGGATTCCCTCAAGC-3'<br>Reverse: 5'- AAGATGGCACCCACGAAG-3'
ATL1	Forward: 5'- GTGGTGGTGACAAACCATTTC-3'<br>Reverse: 5'- CCTCGGAATAGCTTCACAGATT-3'
STIM1	Forward: 5'- CCTCTCTTGACTCGCCATAATC-3'<br>Reverse: 5'- CTTGGAGTAACGGTTCTGGATATAG-3'
GAPDH	Forward: 5'- CCATGTTCGTCATGGGTGTG-3'<br>Reverse: 5'- CAGGGGTGCTAAGCAGTTGG-3'

Supplementary Table 3. Primer sequences used for quantitative PCR.
